# Comparison of 16S ribosomal RNA gene sequence analysis and conventional culture in the environmental survey of a hospital

**DOI:** 10.1186/s40780-017-0074-y

**Published:** 2017-01-19

**Authors:** Akihiro Manaka, Yutaka Tokue, Masami Murakami

**Affiliations:** 1 Department of Clinical Laboratory Medicine, Gunma University Graduate School of Medicine, Maebashi, Gunma Japan; 2 Infection Control and Prevention Center, Gunma University Hospital, 3-39-15 Showa-machi, Maebashi, Gunma Japan

**Keywords:** 16S ribosomal RNA, *Acinetobacter* sp, Hospital environment, Infection control, Nosocomial infection

## Abstract

**Background:**

Nosocomial infection is one of the most common complications within health care facilities. Certain studies have reported outbreaks resulting from contaminated hospital environments. Although the identification of bacteria in the environment can readily be achieved using culturing methods, these methods detect live bacteria. Sequencing of the 16S ribosomal RNA (16S rRNA) gene is recognized to be effective for bacterial identification. In this study, we surveyed wards where drug-resistant bacteria had been isolated and compared conventional culture methods with 16S rRNA gene sequencing methods.

**Methods:**

Samples were collected using sterile swabs from two wards (northern and southern) at Gunma University Hospital contaminated by *Acinetobacter* sp.. We extracted DNA directly from the swabs. Following extraction, the DNA was amplified using polymerase chain reaction (PCR). The PCR products were cloned using the plasmid vector. The plasmid DNA were sequenced, and identification were performed using database. 16S rRNA gene sequence analyses were compared conventional culture methods.

**Results:**

In the northern ward, *Acinetobacter* sp. was detected from only two of 14 samples using the culture method. In contrast, 16S rRNA gene sequencing analysis detected *Acinetobacter* sp. from seven of 14 samples. Drug-resistant *Acinetobacter* sp. was isolated from bathrooms of the southern ward and was detected from four of seven samples using the culture method in comparison with six of seven samples by 16S rRNA gene sequencing analysis.

**Conclusions:**

Molecular biological analysis showed a higher sensitivity to detect specific bacteria and detected a greater number of species than the culture method. Our results suggest that 16S rRNA gene sequencing analysis is useful to identify range of contamination which were not found in conventional culture method. When a nosocomial outbreak cannot be adequately controlled, molecular biological analysis may serve as a useful tool for environmental surveys in hospitals.

## Background

Health care facilities provide an environment conducive to exposure and transmission of bacteria. Nosocomial infections represent one of the most common complications within health care facilities. At least 5–10% of patients admitted to acute care hospitals acquire an infection during hospitalization [1]. Drug-resistant bacteria cause nosocomial infection in patients who have received several courses of antibiotics and those with immune deficiency associated with malignancies or transplants [2]. Some studies have reported outbreaks resulting from the contaminated hospital environment [3, 4]. Although culture methods readily identify environmental bacteria, they only detect live bacteria. Previous research showing that <1% of bacterial species within a given environment are culturable suggests that true diversity is overlooked by studies relying only on culturing [5, 6]. The gene target most commonly used for bacterial identification is the 16S rRNA gene, which is an approximately 1500-bp gene encoding a portion of the 30S ribosomal subunit [7]. 16S rRNA gene sequence analysis is not only widely used as a taxonomic tool but also recognized as an effective reference method for bacterial identification [8]. Samples for 16S rRNA sequence analysis have been expanded from the bacteria in environments such as the oceans and soils to clinical settings. [9–12]. Studies of microbial diversity in child care facilities using culture-independent methods have suggested that knowledge of microbial diversity facilitates better understanding of public health risks in these environments [13]. Therefore, to obtain accurate information regarding contaminated environments within health care facilities, comprehensive reevaluation of microorganisms using culture-independent methods is required.

The number of patients testing positive for *Acinetobacter* sp. has increased, suggesting that outbreaks of *Acinetobacter* sp. infections have occurred. We surveyed the wards where *Acinetobacter* sp. were isolated using culture and culture-independent methods. We aimed to compare these methods and obtain useful information on infection control.

## Methods

### Environmental survey of a hospital

Samples were collected from environments contaminated by *Acinetobacter* sp. at Gunma University Hospital (northern and southern wards). Sampling places were determined from the clinical course of the patients who isolated *Acinetobacter* sp. in consideration of estimated route of infection and range of contamination. As a result, we surveyed the patient’s rooms of the northern ward and the bathroom of the southern ward. Environmental samples were collected twice using sterile swabs. One of the sterile swabs were applied to Sheep blood agar (Eiken Chemical Co., Tochigi, JP) and chocolate agar plates (Becton, Dickinson and Company, Franklin Lakes, NJ), which were incubated of aerobic and anaerobic conditions (5% carbon dioxide gas) 48 h at 35 °C. The following day, grown bacterial colonies were identified using kits as necessary. Other of sterile swabs were then soaked in sterile DNase-free 1.5 mL microcentrifuge tubes containing 200 μL lysis buffer (25 mM Tris–HCl p H 8, 2.5 mM ethylenediaminetetraacetic acid (EDTA), and 1.2% Triton X-100). The swaps were applied to various environmental surfaces in each ward.

### Preparing lysates/purification of genomic DNA

We used a protocol for preparing lysates to lyse gram-positive bacterial cells using the Invitrogen PureLink Genomic DNA Kit (Invitrogen^TM^, Carlsbad, CA).

Following centrifugation of the samples at 10,000 × g for 2 min, the supernatant was discarded and 180 μL lysis buffer containing fresh lysozyme was added to obtain a final lysozyme concentration of 20 mg/mL.

The reaction mixtures tubes were incubated at 37 °C using a heat block for 30 min. Subsequently, 20 μL Proteinase K (PureLink Genomic DNA Kit, Invitrogen^TM^, Carlsbad, CA) and 200 μL PureLink Genomic Lysis/Binding Buffer (PureLink Genomic DNA Kit, Invitrogen^TM^, Carlsbad, CA) were added to the tubes and mixed well by brief vortexing. The tubes were incubated at 55 °C using a heat block for 30 min. A volume of 200 μL 100% ethyl alcohol (EtOH) was then added to the tubes and mixed well by vortexing for 5 s to yield the homogenous solutions.

All reaction mixtures tubes were subjected to a purification procedure using the Invitrogen PureLink Genomic DNA Kit according to the manufacturer’s instructions. Purified genomic DNA was eluted in 50 μL PureLink Genomic Elution Buffer (10 mM Tris–HCl pH 9.0, 0.1 mM EDTA). Following extraction, the purified genomic DNA was confirmed using a NanoDrop ND-1000 spectrophotometer (NanoDrop Technologies, Wilmington, DE) at 260 nm.

### PCR amplification of purified genomic DNA

16S rRNA genes were amplified from purified genomic DNA using the universal primer 8 F(5’-AGAGTTTGATCCTGGCTCAG-3’) and 805R (5’-GACTACCAGGGTATCTAATCC-3’). The primers react with highly conserved regions of the bacterial 16S rRNA gene to provide PCR products of approximately 800 bp. The PCR products from amplification have been shown to be particularly useful for database analysis and identification of bacterial sequences [14].

A Takara Taq Hot-Start kit (Takara Bio Inc., Shiga, JP) was used for amplifications. PCR reactions were performed in a total volume of 50 μL, consisting of 0.25 μL Takara Taq DNA polymerase Hot-Start (5 units/μL), 5 μL 10 × PCR buffer with MgCl_2_, 4 μL deoxynucleotide (dNTP) mixture (2.5 mM each), 1.5 μL each of primer (20 μM), 5 μL of sample DNA as a template, and sterilized distilled water.

Cycling conditions included a initial denaturation at 95 °C for 5 min, followed by 28 cycles of 30 s 95 °C denaturation, 30 s annealing at 55 °C, and 1.5 min elongation at 72 °C, followed by a final extension of 10 min at 72 °C. Afterwards, we confirmed bands of the PCR products that corresponded to the 800 bp using 1.5% agarose gel.

PCR products were subjected to a purification procedure using the QlAquick PCR Purification Kit (QIAGEN, Valencia, CA) according to the manufacturer’s instructions. Purified PCR products were eluted in 50 μL sterilized distilled water. Following extraction, the purified PCR products were quantified using a NanoDrop ND-1000 Spectrophotometer (NanoDrop Technologies, Wilmington, DE).

### Cloning reaction and transformation

The purified PCR products were cloned using the TOPO TA Cloning Kit for Sequencing (Invitrogen^TM^, Carlsbad, CA) according to the manufacturer’s instructions.

Cloning reactions were performed in a total volume of 6 μL, consisting of 0.5 to 4 μL purified PCR products, 1 μL salt solution (1.2 M NaCl, 0.06 M MgCl_2_), 1 μL TOPO vector (10 ng/μL plasmid DNA), and sterilized distilled water. The cloning reaction mixture tubes were mixed gently and incubated for 10 min at room temperature, following which they were placed on ice. Transformation using Takara *Escherichia coli* DH5 α competent cells (Takara Bio Inc., Shiga, JP) was performed according to the One Shot® chemical transformation protocol of the TOPO TA Cloning Kit for sequencing. The transforming reaction mixture tubes were then incubated on ice for 5 min. Next, the cells were heat shocked for 30 s at 42 °C without shaking, and the tubes were immediately transferred to ice. A volume of 250 μL S.O.C. medium (2% tryptone, 0.5% yeast extract, 10 mM NaCl, 2.5 mM KCl, 10 mM MgSO_4_, 10 mM MgCl_2_, and 20 mM glucose) was then added. The tubes were tightly capped and shaken at 200 rpm horizontally at 37 °C for 1 h. The suspensions of competent transformed *E. coli* were then spread at volumes of 10 and 50 μL on the prewarmed agar plates containing ampicillin (Invitrogen^TM^, Carlsbad, CA) and were incubated overnight at 37 °C. By the following day, colonies had grown from the agar plates, and 15 colonies were randomly selected and cultured overnight at 37 °C in Falcon 2059 tubes (Becton, Dickinson and Company, Franklin Lakes, NJ) in 1 mL of lysogeny broth (LB) medium (Fisher Biotech, Fair Lawn, NJ) containing 100 μg/mL ampicillin.

### Isolation of plasmid DNA

LB medium cultured overnight was collected into sterile DNase-free 1.5 mL microcentrifuge tubes. The tubes were then centrifuged at 12,000 × g for 2 min, with the pellets used for further analysis and the supernatant discarded. The plasmid DNA in the pellets was isolated using the PureLink Quick Plasmid DNA Miniprep Kit (Invitrogen^TM^, Carlsbad, CA) according to the manufacturer’s instructions. Purified plasmid DNA was eluted in 75 μL sterilized distilled water.

### Sequencing and identification

The primer T3 (5’-ATTAACCCTCACTAAAGGGA-3’) was used for sequencing. Sequencing of purified plasmid DNA was completed using the Applied Biosystems 3730xl DNA sequencer. Identifications were performed using the Basic Local Alignment Search Tool (BLAST), with a similarity cut-off of 99%.

## Results

### Environmental survey of the northern ward

*Acinetobacter* sp. was detected from only two of 14 samples using the culture method. In contrast, using 16S rRNA gene sequence analysis, *Acinetobacter* sp. was detected from seven of 14 samples. Thus, positive results were obtained for five samples by only the 16S rRNA gene sequence analysis.

Table 1 shows the results of the environmental survey of the northern ward comparing the 16S rRNA gene sequence analysis and the culture method.

**Table 1:** 16S rRNA gene sequencing analysis and culture method results for the environmental survey of the northern ward

The northern ward	Samples	16S rRNA analysis	Sequence length^1)^	%, Identity^2)^	Culture method
Nurses’ station	System of urinal collection	*Corynebacterium* sp. *Propionibacterium* sp. *Neisseria* sp. *Staphylococcus* sp. *Actinomyces* sp.	880 810 786 818 801	100 99 99 100 99	(−)
	Personal computer	***Acinetobacter*** **sp.** *Staphylococcus* sp. *Corynebacterium* sp. *Propionibacterium* sp.	810 797 801 794	99 99 99 100	(−)
	Faucet	***Acinetobacter*** **sp.** *Serratia* sp. *Staphylococcus* sp.	884 790 806	99 99 99	(−)
	Outlet	***Acinetobacter*** **sp.** *Stenotrophomonas* sp.	816 791	99 99	*Enterobacter cloacae* *Stenotrophomonas maltophilia*
Patient’s room 1	Fence of the bed	*Staphylococcus* sp. *Propionibacterium* sp. *Corynebacterium* sp.	884 802 817	99 99 99	CNS
	Aspirator	***Acinetobacter*** **sp.** *Corynebacterium* sp. *Staphylococcus* sp. *Neisseria* sp. *Bacteroides* sp. *Prevotella* sp. *Actinomyces* sp.	802 794 793 798 819 800 786	99 99 99 100 99 99 99	(−)
	Trash box	*Staphylococcus* sp. *Stenotrophomonas* sp. *Corynebacterium* sp.	805 789 814	99 99 99	CNS *Klebsiella pneumoniae* *Asperugirus* sp.
Patient’s room 2	Fence of the bed	***Acinetobacter*** **sp.** *Corynebacterium* sp. *Klebsiella* sp. *Enterocococcus* sp. *Fusobacterium* sp. *Lactobacillus* sp. *Prevotella* sp.	823 820 746 738 805 812 794	99 99 99 99 99 99 99	(−)
	Intubation tube	***Acinetobacter*** **sp.** *Corynebacterium* sp. *Staphylococcus* sp. *Stenotrophomonas* sp. *Fusobacterium* sp. *Lactobacillus* sp.	805 882 797 806 802 802	99 99 99 99 99 99	***Acinetobacter baumannii***
Patient’s room 3	Fence of the bed	*Staphylococcus* sp. *Corynebacterium* sp. *Actinomyces* sp.	844 889 820	99 99 99	CNS
	Intubation tube	***Acinetobacter*** **sp.** *Staphylococcus* sp. *Stenotrophomonas* sp.	804 836 801	99 99 99	***Acinetobacter lwoffii*** CNS
Patient’s room 4	Outlet	*Stenotrophomonas* sp.	803	99	*Aeromonas hydrophia* *Stenotrophomonas maltophilia*
	Curtain	*Staphylococcus* sp. *Streptococcus* sp. *Neisseria* sp. *Corynebacterium* sp. *Escherichia* sp. *Fusobacterium* sp.	773 771 801 798 803 800	99 99 99 99 99 99	(−)
Dirty utility room	Urinal surface	*Escherichia* sp.	823	99	(−)

*CNS* coagulase-negative staphylococci

^1)^Length of sequence used in the Basic Local Alignment Search Tool (BLAST) search

^2)^Based on BLAST alignment

*Acinetobacter* sp. was not detected using the culture method in the nurses’ station; however, 16S rRNA gene sequence analysis provided positive results for three samples: the personal computer, faucet, and outlet samples. In patients’ rooms, *Acinetobacter* sp. was detected from only two samples using the culture method. These two samples were obtained from intubation tubes. In contrast, using 16 s rRNA gene sequence analysis, four samples containing the different samples from the culture method were identified as positive: the intubation tubes, the aspirator, and railings of a bed. Several samples showed different results between the 16S rRNA gene sequence analysis and the culture method. The various bacteria identified by 16S rRNA gene sequence analysis but not the culture method were collected from dry environmental surfaces. Six samples presented anaerobes by 16S rRNA gene sequence analysis. While 16S rRNA gene sequence analysis could not detect *Aspergillus*, these fungi were detected by the culture method.

### Environmental survey of the bathroom of the southern ward

Drug-resistant *Acinetobacter* sp. were detected from four of seven samples using the culture method. These four samples were collected from the faucet, bath chair, handrail, and the drain outlet of the bathroom. In contrast, 16S rRNA gene sequence analysis detected *Acinetobacter* sp. from six of seven samples. These six samples were collected from the faucets, bath chair, the bathtub drain, the handrail, and the drain outlet of the bathroom. In particular, two samples were detected only by 16S rRNA gene sequence analysis.

Table 2 shows the results of the environmental survey of the bathroom of the southern ward comparing the 16S rRNA gene sequence analysis and the culture method. Several samples showed different results between the 16S rRNA gene sequence analysis and the culture method. 16S rRNA gene sequence analysis detected uncultured bacteria from the shower head of the bathroom. Fungal strains were not detected using the 16S rRNA gene sequence analysis, but were detected by the culture method.

**Table 2:** 16S rRNA gene sequencing analysis and culture method results for an environmental survey of the bathroom of the southern ward

Samples	16S rRNA analysis	Sequence length^1)^	%, Identity^2)^	Culture method
Shower head	*Sphingomonas* sp. *Methylobacterium* sp.	826 829	99 100	(−)
Faucet 1	***Acinetobacter*** **sp.**	721	99	Fungus
Faucet 2	***Acinetobacter*** **sp.**	661	99	***Acinetobacter baumannii***
Bath chair	***Acinetobacter*** **sp.**	803	99	***Acinetobacter baumannii*** *Stenotrophomonas maltophilia*
Outlet of the bathtub	***Acinetobacter*** **sp.**	804	100	*Pseudomonas aeruginosa*
Handrail	***Acinetobacter*** **sp.**	760	99	***Acinetobacter baumannii***
Outlet of the bathroom	***Acinetobacter*** **sp.** Non Tb *Mycobacterium* sp.	714 736	100 99	***Acinetobacter baumannii***

*Tb* tuberculosis

^1)^Length of sequence used in Basic Local Alignment Search Tool (BLAST) search

^2)^Based on BLAST alignment

## Discussion

The spectrum of organisms causing nosocomial infections is under continual flux. From the 1970s through to 2000, the spectrum of nosocomial pathogens shifted from Gram-negative to Gram-positive organisms, and *Candida* spp. emerged as a major problem [15]. More recently, multidrug-resistant Gram-negative rods have become increasingly prevalent in many hospitals [1]. *Acinetobacter* sp. is rapidly emerging as a pathogen in health care settings, where it results in infections that include bacteremia, pneumonia, meningitis, urinary tract infection, and wound infection [16]. *Acinetobacter* sp. is able to survive for long periods on dry surfaces, and this ability to tolerate desiccation may contribute to its persistence in hospitals [17]. Treatment options are severely limited, and carbapenems are the agents of choice for treating most drug-resistant infections [18]. Unfortunately however, carbapenem-resistant *Acinetobacter* isolates are increasingly reported worldwide [18]. Carbapenem-resistant *Acinetobacter* infections have an extremely high crude mortality rate and occur most frequently in severely ill patients [18].

In the present study, 16S rRNA gene sequence analysis showed higher sensitivity to detect specific bacteria than the usual culture method. In addition, some bacteria strains that were not detected using the culture method were detected in the environmental samples.

In the case of an outbreak, environmental surveillance in a hospital is usually performed using the culture method [3, 4, 19, 20]. The culture method requires an appropriate culture media and culture condition that accept target bacteria. In addition, some bacteria require an extended time for isolation; thus, a delay may be experienced in obtaining the preliminary results. In the present study, the culture method was not able to detect five of the seven samples that the 16S rRNA gene sequencing analysis identified as positive. This is because the samples did not obtain viable bacteria. On the other hand, 16S rRNA gene sequence analysis has been used to identify novel and emerging pathogens and to define complex microbial communities [21]. This analysis is especially valuable for detecting bacteria that are slow growing, biochemically inert or variable, and fastidious. In addition, 16S rRNA gene sequence analysis has enhanced our understanding of previously unrecognized, often opportunistic pathogens [22]. According to recent studies, this molecular biology analytical method has been used in the search of pathogens of infectious diseases, reporting a higher rate of detection than the usual culture method [9, 23]. Indeed, 16S rRNA gene sequence analysis provides a comprehensive assessment of microbial diversity compared with culturing alone, and is an excellent complement to the culturing approaches. In the present study, anaerobic bacteria were detected in six samples. The inconsistencies between the 16S rRNA gene sequence analysis and the culture method observed in the present study suggest that the culture method alone may not fully express precise bacterial information in the contaminated environment of a hospital. To our knowledge, this is the first report that 16S rRNA gene sequence analysis is applicable to infection control by detection of contaminated area undetectable by usual culture method. This analysis might become a useful tool for environmental surveys in hospitals in cases of nosocomial outbreaks.

There are several limitations associated with the present study. First, closely related species might be difficult to distinguish using 16S rRNA gene sequence analysis, and the identification of bacteria to a species level might be inaccurate. Second, the antimicrobial susceptibility of bacteria is not obtained by 16S rRNA gene sequence analysis. Third, fungal strains could not be detected using 16S rRNA gene sequence analysis. Finally, the number of clones analyzed in the present study was 15 per library, which might be insufficient to detect very small fractions of the library.

## Conclusions

Because 16S rRNA gene sequence analysis is more sensitive to detect in environmental samples, it is possible to identify range of contamination which were not found in conventional culture method. This analysis might become a useful tool for environmental surveys in hospitals in cases of nosocomial outbreaks.

## Abbreviations

16S rRNA: 16S ribosomal RNA
BLAST: Basic local alignment search tool
dNTP: Deoxynucleotide
EtOH: Ethyl alcohol
EDTA: Ethylenediaminetetraacetic acid
LB: Lysogeny broth
PCR: Polymerase chain reaction
